# The Impact on Emergency Department Utilization and Patient Flows after Integrating with a General Practitioner Cooperative: An Observational Study

**DOI:** 10.1155/2013/364659

**Published:** 2013-10-03

**Authors:** W. A. M. H. Thijssen, M. Wijnen-van Houts, J. Koetsenruijter, P. Giesen, M. Wensing

**Affiliations:** ^1^Catharina Hospital, Michelangelolaan 2, P.O. Box 1350, 5602ZA NB Eindhoven, The Netherlands; ^2^IQ Scientific Institute for Quality of Healthcare (IQ Healthcare), Radboud University Nijmegen Medical Centre, IQ P.O. Box 9101, Healthcare 114, 6500HBGLO Nijmegen, The Netherlands; ^3^Emergency Department, Catharina Hospital, Eindhoven P.O. Box 1350, 5602ZA NB Eindhoven, The Netherlands

## Abstract

*Introduction*. A new model, an emergency care access point (ECAP) for after-hours emergency care, is emerging in The Netherlands. This study assessed the effect on emergency department (ED) utilization and patient flows. *Methods*. Routinely recorded clinical ED patient data, covering a six-year period, was collected. Segmented regression analysis was used to analyze after-hours changes over time. *Results*. 59.182 patients attended the ED before the start of the ECAP and 51.513 patients after, a decrease of 13%. Self-referred ED patients decreased 99.5% (OR 0.003; 95% CI 0.002–0.004). Referred patients increased by 213.4% and ED hospital admissions increased by 20.2%. A planned outpatient follow-up increased by 5.8% (OR 1.968 95% CI 1.870–2.071). The latter changed from fewer contacts to more contacts (OR 1.015 95% CI 1.013–1.017). Consultations at the regional genereral practitioner cooperative (GPC) increased by 26.0% (183.782 versus 232.246). *Conclusion*. ECAP implementation resulted in a decrease in ED utilization, a near absence of self-referring patients, and a higher probability of hospital admission and clinical follow-up. This suggests either an increase of ED patients with a higher acuity or a lower threshold of admitting referred patients compared to self-referred patients. Overall, increased collaboration with after-hours primary care and emergency care seemed to optimize ED utilization.

## 1. Introduction

Organizing after-hours care is an important challenge in many countries. After-hours care is provided between 5 p.m. and 8 a.m., on weekends and national holidays. In most western countries emergency departments (EDs) are confronted with overcrowding, while general practitioners (GPs) are not all easily accessible, especially after hours. Differences in national healthcare systems have a noticeable effect on redirecting patients to primary care services. In the United States, studies show an increase of 23–27% in ED visits between 1997 and 2008 [1, 2]. Simultaneously, delivering primary care access after hours decreased from 40% of the GPs in 2006 to 29% in 2009 and different models exist [3–5]. In western Europe, the GP plays a significant role in providing after-hours care, with 77% of the GPs in Italy, 89% in the UK, and 97% in The Netherlands providing after-hours arrangements [3]. Across Europe, different models of after-hours primary care exist, varying from local rotation groups to large General Practitioner Cooperatives (GPCs) [6, 7]. Despite good primary care access, high and rising ED visits are also an issue in Europe [8].

**Figure 1:**
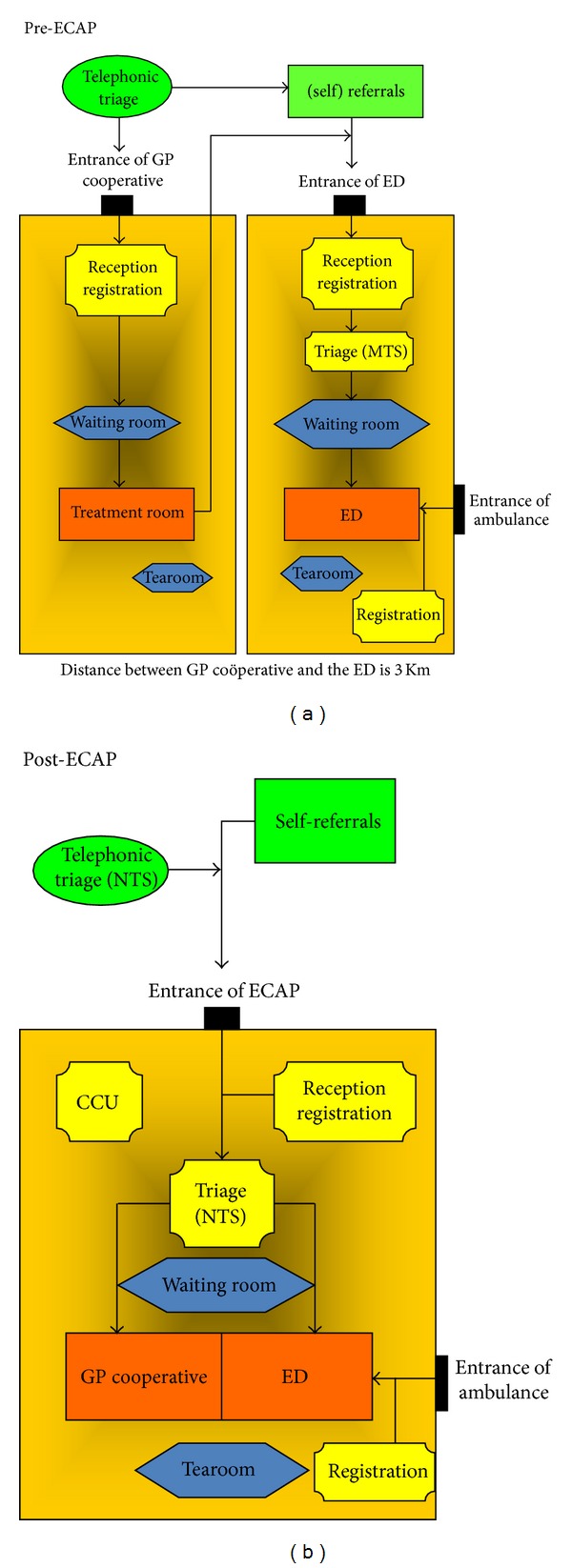
The GP can order blood tests during ECAP hours and order X-rays until 10 p.m., without referring the patient to the ED. This is similar to office hours.

In the Dutch healthcare system, precise numbers are lacking, but an estimated average of 1.9–2.1 million patients visited the ED per year between 2004 and 2008, with 135 treatments per 1000 inhabitants per year [9]. The GP is the coordinator of access to the hospital specialist for the majority of emergency care. Primary care is provided 24 hours a day, 7 days a week. It is always free at the point of entry, while patient copayments are required for visits to the ED. During office hours, GPs provide patient care in their office-based practices, including emergency care. Patients can contact their GP through the practice phone number for a telephone advice, a consultation or a home visit. Self-referred patients, who present to the ED during office hours and who are eligible for a GP consultation, are given the option to contact their own GP for an appointment. If they insist on receiving medical care in the ED, they are registered for treatment in the ED. The organization of after-hours primary care has changed in recent years from rota groups to GPCs, mainly to reduce GPs' workload and also to improve the quality of after-hours care. Since 2000, large-scale GPCs have emerged in the Netherlands, with around 130 GPCs serving the Dutch population of nearly 17 million inhabitants [6, 9, 10]. Nonetheless, there is a rise in self-referrals to the ED, because patients can still choose to visit the ED directly and bypass the GPC. To enhance efficiency, decrease overcrowding and costs of after-hours care by redirecting the patient that does not need hospital care to the GP, an organizational model has been proposed that integrates the GPC and the ED into a colocation with one emergency care access point (ECAP), Figure 1.

At this integrated emergency care access point (ECAP), triage will determine whether patients will be seen by a GP or by a physician in the ED. Only a few integrated models exist at this point. Influenced by local successes, several GPCs and EDs are planning to integrate in the near future. Despite the growing interest, little is known of the ECAP effect on flow of ED patients and their planned follow-up. Several small studies over a short period show different results varying from no flow change to a shift of 15–53% of patients from the ED to the GPC [11–20]. 

In our study, we took advantage of a natural organizational change, which occurred in the south east region of The Netherlands. The local GPC integrated with the ED of an inner city hospital during after-hours care, creating a colocation with one emergency care access point. 

 The objective of this study was to examine the effect of an ECAP on ED utilization and planned patient follow-up. We expected that a large part of the self-referrals could be redirected to the GPC, leading to a decrease in ED utilization. Furthermore we expected an increase of hospital admissions and out-patient clinical follow-up, since the GP is more likely to refer patients to the colocated ED then to another hospital. 

## 2. Materials and Methods

We conducted a longitudinal observational study in an integrated after-hours care model between an urban emergency department (ED) and a general practitioner cooperative (GPC) in the southeast region of The Netherlands, serving a population of around 325.000 people. In this model, the ED and the GPC, who formerly worked separately, now integrated forming a colocation with one emergency care access point (ECAP), (Figure 1). To determine the ECAP effect on the trend of ED patient flows, we compared routinely collected ED data three years before and three years after the implementation of the ECAP. Since there is no colocation during office hours, meaning that patients can bypass the GP, we also collected ED data during non-ECAP hours to see overall changes in trends. We hypothesized an increase in GPC patients and, therefore, also collected GPC data to analyze changes before and after the ECAP implementation. The medical ethical committee of the hospital granted institutional review board exemption.

### 2.1. Organizational Changes

Before the study, the ED and the GPC were located in different areas in the city, 3 kilometres apart. Although patients were encouraged to contact the GPC first, they could choose to visit the ED at their own initiative. The collaboration of the ED and the GPC to form an ECAP with one triage system in December 2008 changed this. It meant a difference in the routing of the patient after-hours because the patient could no longer willingly bypass the GP (Box 1). The ECAP is open from 5 p.m. till 8 a.m. during weekdays, the weekend, and national holidays. As before the change, patients are encouraged to phone the GPC first via a regional telephone number, when they seek medical help during ECAP hours. The after-hours change into an ECAP and the ways to contact the GP were promoted by flyers in the ED, GPC, and in waiting rooms of both the hospital and all regional GPs and by advertisements in local newspapers, prior to the implementation. A call center for the regional telephone number is colocated with the ECAP and is manned by telephone operators who are trained in using The Netherlands triage system (NTS) to determine patient urgency. NTS is a triage system that is developed and validated in The Netherlands to work for the ambulance services, GPCs, and EDs [21]. It determines the urgency, type of medical advice (consult, home visit, or telephone advice) and type of healthcare provider (ambulance, GP, or ED). A GP at the call center supervises all phone calls. Depending on the complaint and telephone triage outcome, patients will either receive a telephone advice, an appointment with a GP at the ECAP, a GP home visit or they will be directly referred to the ED. If necessary, the ECAP can send out an ambulance as well. Independently of the ECAP, there is also the national emergency phone number, 112, that patients can phone 24/7, to request an ambulance. Self-referred patients who do not phone and turn up at the ECAP are registered and triaged with NTS by a trained triage nurse. Depending on their triage outcome, patients either receive a scheduled appointment at the GPC or are directly referred to the ED. The GP supervises the triage nurse; therefore, all patients that are directly referred to the ED after triage are registered as GP referrals. Patients who have been treated by a hospital physician either in the outpatient clinic or through a hospital admission, within three months prior to presenting at the ECAP, were automatically referred to the ED and registered as a return visit. This was a local agreement between GPs and hospital physicians prior to the start of the ECAP. The GP can request simple blood tests 24/7 and X-rays until 10 p.m., without having to refer the patient to the ED, similar to office hours.

**Box 1:**
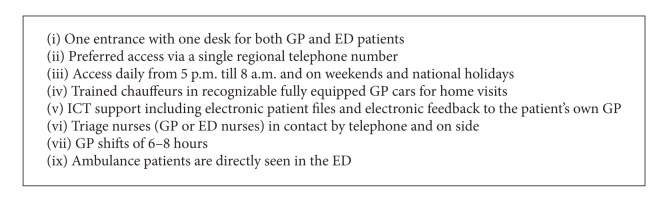
Features of an integrated GPC and ED (ECAP).

### 2.2. Study Setting

The studied ED is the only ED in the city and is situated just outside the city centre, serving the major part of the city and the northern region outside the city. There used to be another hospital with a small ED that closed down in August 2008, during the time frame of our study, three months before the study intervention. 

The GPC is a large cooperation that has three locations in the region, serving 510.000 people. Two locations are outside the city. The studied GPC in the city was initially located three kilometers from the ED before integrating. 

### 2.3. Measures

For the ED, we gathered data from the CS-EZIS system, Chipsoft BV, which is a computerized system routinely used for real time management of patients. Every patient that presents to the ED is registered in this system. We collected clinical data of the ED, covering a six-year period from 2006 to 2012, three years before and three years after the start of the emergency care access point (ECAP). Because the ECAP started on 1 December 2008, we considered this a transition month and did not include this month in our data. For comparison reasons, we therefore also excluded data from December 2011, making the pre ECAP period and the post ECAP period equal, namely 35 months.

For the ECAP hours, we collected patient characteristics as gender and age. For patient origin, the EZIS system has 7 categories: self-referral, GP referral, ambulance referral, return visit, outpatient clinical referral, radiology referral (e.g., a proven fracture), and referrals from another hospital. We also collected data about the next clinical step after the ED visit, where the EZIS system also has 7 categories: no follow-up, GP follow-up, outpatient clinical follow-up, hospital admission, transfer to another hospital, left without being seen, and either died in the ED or brought in dead. 

We also collected absolute ED patient numbers from non-ECAP hours to look at the overall trend of ED visits. Finally we collected regional GPC data on the number of ECAP visits, telephone advice, and home visits before and after the start of the ECAP.

### 2.4. Data Analysis

**Figure 2:**
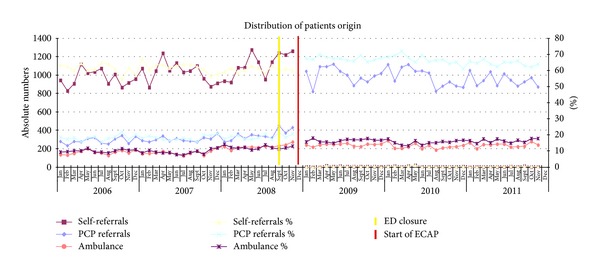
Distribution of patients origin. For visual reasons the percentage of revisits, referrals from outpatient clinics or other hospitals is not shown in this figure.

**Figure 3:**
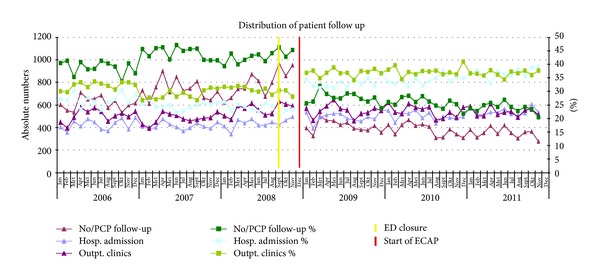
Distribution of patient follow-up. For visual reasons the percentage of deceased in the ED and transfer to another hospital is not shown in this figure. These percentages are very low and remained unchanged.

For the comparison of descriptive characteristics of patients before and after the start of the ECAP, we used the chi-square test. We calculated numbers and percentages. A logistic segmented regression analysis was performed to test the changes after implementation of the ECAP as shown in Figures 2 and 3. This method estimates separately the change over time before the intervention, the direct effect of the intervention itself, and the change over time after the intervention. *P* < 0.05 was considered significant. Outcome variables were self-referrals versus non-self-referrals and an outpatient clinical follow-up contact versus no clinical follow-up contact (the combined categories of no follow-up contact and a GP follow-up contact). The regression model controlled for the effect of age and gender. We used SPSS 19.0 for our statistical analysis.

The following categories were used: hospital admission, outpatient clinical follow-up and no outpatient clinical follow-up (GP follow-up or no follow-up combined). Two periods were distinguished: before the start of the ECAP (1 January 2006 till 1 December 2008) and after the start of the ECAP (1 January 2009 till 1 December 2011), 35 months in each period. 

## 3. Results

In the non-ECAP hours, a total of 94.778 patients attended the ED, 44.190 in the period before the start of the ECAP and 50.588 after the start, an increase of 14%. During ECAP hours over the same period, a total of 110.696 patients attended the ED, 59.182 ED patients before the start of the ECAP and 51.513 patients after the start, an overall decrease of 13%. During ECAP hours, the majority in both groups, pre- and post-ECAP, were males, 54.5% and 52.1%, respectively. The mean patient age shifted from 39 to 44 years old. The percentage of total ED patients in younger age groups (0–49 years) decreased and older age groups (50 and older) increased with 9.3%. The group of 65 and older increased by 16.2%. The majority of patients presented to the ED in the evening between 5 p.m. and 11 p.m. (51.7% and 49.6%). All changes were significant (Table 1).

Figure 2 shows that in the post-ECAP period, the number of ED self-referrals decreased by 99.5% (36.139 versus 181), GP referrals increased by 213.4% (10.876 versus 34.089) and the number of patients brought in by ambulance increased by 3.2% (6.244 versus 8.222). The logistic segmented regression analysis showed a slight decrease in self-referrals before the introduction of the ECAP (OR 0.997; 95% CI 0.995–0.999). After the intervention, there was an instant, considerable decrease in self-referrals (OR 0.003; 95% CI 0.002–0.004). 

After the start of the ECAP, both hospital admissions, 14.854 versus 17.827, as well as outpatient clinical follow-up, 18.003 versus 19.047, increased with 20.2% and 5.8%, respectively. There was a decrease of 46.4% in ED patients referred back to their GP for follow-up or who did not need follow-up at all, 24.896 versus 13.342 (Figure 3). The regression analysis showed that the number of outpatient clinical follow-up contacts and hospital admissions decreased before the introduction of the ECAP (OR 0.993; 95% CI 0.991–0.995). Directly after the introduction of the ECAP, the number of follow-up contacts increased (OR 1.968; 95% CI 1.870–2.071) and the trend changed from less contact to more contacts (OR 1.015; 95% CI 1.013–1.017). All changes were significant.

Table 2 shows the effect of the closing of the other city's ED 3 months prior to starting the ECAP. There is an average increase of 430 ED patient visits per month (31.9%). There was no noticeable change in percentage of self-referrals (59.2% and 60.1%) or GP referrals (18.4 and 20.2%). The increasing trend of no/GP follow-up (5.2% and 1.8%) and decreasing trend of hospital admission (2.6% and 2.0%) remained unchanged after the closing.

Table 3 shows that after implementing the ECAP, the total number of patients having contact with the GPC increased by 30% (330.162 versus 412.545). Patients receiving a consult at the regional GPC increased by 26.0% (183.782 versus 232.246) and patient home visits decreased by 14.3% (33.618 versus 28.818).

## 4. Limitations

With the implementation of the ECAP, the triage system changed from the Manchester triage system (MTS) to The Netherlands triage standard (NTS), and both systems are not comparable. We therefore had to look at hospital admissions and outpatient clinical follow-up for comparison of patients acuity. 

Three months before the organizational change, the only other emergency department in the city closed. This led to a shift in patients spreading out over other hospitals in the region, including the studied ED, and this might have affected total patient numbers but should not have had an effect on trends.

The observational design implies that factors outside the ECAP may have contributed to the results. The study used data that were retrospectively analyzed. This study took advantage of a naturally occurring change in a large geographical area, but its relevance for other settings has to be considered.

## 5. Discussion

The implementation of an ECAP led to a decreased ED utilization, with almost no self-referring patients and an increase in ED patients with a hospital admission or planned outpatient follow-up. These findings suggest that fewer but more patients with a higher acuity attended the ED after the start of the ECAP.

This is one of the first long-term studies to observe the changes in attending ED patients after implementing an ECAP, so it is difficult to compare it with available literature. We found a patient reduction of 13% after the start of the ECAP. It is plausible to assume that patient numbers would have followed the same trend after hours as during office hours (14% increase), had the ECAP not been implemented. The total effect of the ECAP would thus be a decrease of 27% of patients attending the ED. Earlier pilot studies, with short follow-up periods, found a shift of low acuity patients, mostly self-referrals, from the ED to the GPC varying from 15% to 53% [13–21]. Although we could not compare triage outcome, our data supports these findings, since the overall ED patient decrease in our study is mostly due to the shift of the younger, male patients, which is typically the low acuity self-referral. In referred patients the increase of 213% we found is much higher compared to other studies [15–21]. Several factors might have contributed to that increase difference, for instance the closing of the other ED, although overall patient numbers do not seem to imply that. It is more likely that, in contrast to most other studies, the patients in our study cannot bypass the GP and visit the ED directly. Furthermore, the protocol to refer every patient, who has been admitted or seen in the outpatient clinic for similar presenting complaints within the previous three months, is likely to lead to higher referral numbers. Decreasing this arbitrary 3-month period could further decrease the number of patients being referred. With respect to patients follow-up, we found an increase of 20.2% in hospital admissions. This differs from the decrease found in a smaller Dutch study and the 0.2% decrease found by Andersson in a study that was also confronted with closing EDs in the region [17, 18]. An explanation could be that starting an integrated ECAP is likely to increase the adherence with more GP referrals to this specific hospital. It is also likely that the GP has a lower threshold to refer patients to the ED next door rather than transporting to another ED. Similarly, the ED physician is likely to have a lower threshold to admit patients being referred by a GP compared to the self-referral. Apart from the increasing aging patient with comorbidities, this trend of increasing hospital admissions could be due to the ongoing development of rapid diagnostic technologies, early treatment availability, and public education campaigns in recognizing heart attacks and strokes [22]. 

Similar to a previous study in The Netherlands, we found an increase in number of patients staying within one setting for follow-up [23]. These previous studies show an increase as well as a decrease of outpatient clinical follow-up after an ED referral [17, 23]. 

Across the world, policy makers are looking for the optimal organization of emergency care [5, 8, 24, 25]. This study suggests that high involvement of primary care providers in emergency care can optimize the efficiency of ED utilization. Patients with serious conditions benefit from the facilities and skills at the ED, while patients with less serious conditions are treated in a primary care setting, reducing ED crowding. The high number of patients receiving planned clinical follow-up might indicate potential for further efficiency gains. Further research is also needed to examine health outcomes in patients attending the ECAP and receiving either primary care or treatment at the ED.

## 6. Conclusion

In summary, the introduction of a regional integrated ECAP in one region in The Netherlands was associated with substantial changes in the flow of patients, including an overall decrease in ED utilization, an almost disappearance of self-referring ED patients, and a higher probability of hospital admission and clinical follow-up at the ED. The latter suggests that either the proportion of patients presenting to the ED with a higher acuity increased or the threshold of admitting referred patient is lower than that of the self-referred patients. The integrated model for emergency care, in which the GP is the first point of contact for patients, works well for The Netherlands. Differences across healthcare systems and after-hours primary care models make it somewhat difficult to predict whether this also applies to other settings, but we suggest that it works well in countries with a well-developed primary care sector. 

## Figures and Tables

**Table 1:** After-hours ED patient characteristics; numbers and percentages of patients, total of 3 years before and 3 years after the start of the ECAP.

	Before		After^II^	
	2006–2008*		2009–2011*	
	*N*	%	*N*	%
Total non-ECAP hours				
94.778	44.190	—	50.588	—
Total ECAP hours				
110.696	59.182	100	51.513	100
Gender				
Male	32.283	54.5	26.843	52.1
Age in years				
1–17	13.873	23.4	10.264	19.9
18–29	10.701	18.1	7.591	14.7
30–49	13.698	23.1	10.674	20.7
50–64	8.604	14.5	8.679	16.8
65–84	10.698	18.1	11.980	23.3
≥85	1.608	2.7	2.325	4.5
Time of visit				
8 a.m.–5 p.m.^†^	16.285	27.5	14.304	27.8
5 p.m.–11 p.m.^‡^	30.575	51.7	25.571	49.6
11 p.m.–8 a.m.^§^	12.322	20.8	11.638	22.5

*Excluding December 2008 and December 2011; ^†^weekend days and national holidays; ^‡^7 days a week; ^§^7 days a week; ^II^all changes are significant after the ECAP using chi-square testing.

**Table 2:** Effect of the closure of another city's ED on patient numbers and percentage on the researched ED.

		Patient	Origin			Follow-up		
			Self-referral	GP referral	Ambulance	No/GP follow-up	Hosp. admissions	Outpatient clinic
∗	ECAP	*N*	%^†^			%^†^		
2006	Before	4697	59.2	19.2	10.6	37.7	27.9	32.5
2007	Before	4902	59.8	18.4	9.8	42.9	25.3	29.5
2008	Before	6193	60.1	20.2	12.0	44.7	22.3	29.5
2009	After	4307	0.5	67.0	17.0	27.1	33.1	37.7
2010	After	4110	0.2	65.3	15.8	25.1	35.6	36.6
2011	After	4399	0.4	62.9	16.9	22.7	37.9	37.1

*Each year contains a three-month period covering September, October, and November. In the before period, the ECAP had not been implemented, and the ED closure in 2008 is the only regional change in the emergency healthcare setting. In the after period, the ECAP is implemented as well. ^†^Mean percentage.

**Table 3:** Patient numbers at the regional GPC before and after the ECAP (after hours).

Total	Pre-ECAP				Post-ECAP				Total
Year	2006	2007	2008	Subtotal	2009	2010	2011	Subtotal	
Telephone advice	33.866	37.485	41.411	**112.762**	52.541	48.481	50,459	**151.481**	**264.243**
GPC consultation	58.062	62.345	63.375	**183782**	77.875	77.812	76,559	**232.246**	**416.028**
Home visit	10.773	11.385	11.460	**33.618**	10.741	9.156	8,921	**28.818**	**72.436**
Total	**102.701**	**111.215**	**116.246**	**330.162**	**141.157**	**135.449**	**135.939**	**412.545**	**752.707**

The regional GPC consists of three GPCs including the studied GPC. Separate GPC data is not available since it is not registered as such.

## References

[1] Tang N, Stein J, Hsia RY, Maselli JH, Gonzales R (2010). Trends and characteristics of US emergency department visits, 1997–2007. *Journal of the American Medical Association*.

[2] Pitts SR, Pines JM, Handrigan MT, Kellermann AL (2012). National trends in Emergency Department Occupancy, 2001–2008: effect of inpatient admissions versus Emergency Department Practise intensity. *Annals of Emergency Medicine*.

[3] Schoen C, Osborn R, Huynh PT, Doty M, Peugh J, Zapert K (2006). On the front lines of care: primary care doctors’ office systems, experiences, and views in seven countries. *Health Affairs*.

[4] Schoen C, Osborn R, Doty MM, Squires D, Peugh J, Applebaum S (2009). A survey of primary care physicians in eleven countries, 2009: perspectives on care, costs, and experiences. *Health Affairs*.

[5] O’Malley SA, Samuel D, Bond A, Carrier E (2012). After-hours care and its coordination with primary care in the US. *Journal of General Internal Medicine*.

[6] Huibers L, Giesen P, Wensing M, Grol R (2009). Out-of-hours care in western countries: assessment of different organizational models. *BMC Health Services Research*.

[7] Grol R, Giesen P, van Uden C (2006). After-hours care in the United Kingdom, Denmark, and the Netherlands: new models. *Health Affairs*.

[8] Majeed A (2012). Redesigning after-hours primary care. *Annals of Internal Medicine*.

[9] Steenwijk PCE, van der Sterren EGS, van Vugt CJ, Samenwerking huisartsenposten en spoedeisende hulp (SEH) Collaboration between GP cooperatives and Emergency Departments.

[10] Thijssen WAMH, Giesen PHJ, Wensing M (2012). Emergency departments in the Netherlands. *Emergency Medicine Journal*.

[11] Giesen P, Smits M, Huibers L, Grol R, Wensing M (2011). Quality of after-hours primary care in the Netherlands: a narrative review. *Annals of Internal Medicine*.

[12] Moll van Charante EP, ter Riet G, Bindels P (2008). Self-referrals to the A&E department during out-of-hours: patients’ motives and characteristics. *Patient Education and Counseling*.

[13] Giesen P, Franssen E, Mokkink H, van den Bosch W, van Vugt A, Grol R (2006). Patient either contacting a general practice cooperative or accident and emergency department out of hours: a comparison. *Emergency Medicine Journal*.

[14] Kool RB, Homberg DJ, Kamphuis HCM (2008). Towards integration of general practitioner posts and accident and emergency departments: a case study of two integrated emergency posts in the Netherlands. *BMC Health Services Research*.

[15] van Uden CJT, Winkens RAG, Wesseling G, Fiolet HFBM, van Schayck OCP, Crebolder HFJM (2005). The impact of a primary care physician cooperative on the caseload of an emergency department: the Maastricht integrated out-of-hours service. *Journal of General Internal Medicine*.

[16] Pickin DM, O’Cathain A, Fall M, Morgan AB, Howe A, Nicholl JP (2004). The impact of a general practice co-operative on accident and emergency services, patient satisfaction and GP satisfaction. *Family Practice*.

[17] Andersson G, Karlberg I (2001). Lack of integration, and seasonal variations in demand explained performance problems and waiting times for patients at emergency departments: a 3 years evaluation of the shift of responsibility between primary and secondary care by closure of two acute hospitals. *Health Policy*.

[18] van Uden CJT, Winkens RAG, Wesseling GJ, Crebolder HFJM, van Schayck CP (2003). Use of out of hours services: a comparison between two organisations. *Emergency Medicine Journal*.

[19] Philips H, Remmen R, van Royen P (2010). What’s the effect of the implementation of general practitioner cooperatives on caseload? Prospective intervention study on primary and secondary care. *BMC Health Services Research*.

[20] O’Kelly FD, Teljeur C, Carter I, Plunkett PK (2010). Impact of a GP cooperative on lower acuity emergency department attendances. *Emergency Medicine Journal*.

[21] van Uden CJT, Crebolder HFJM (2004). Does setting up out of hours primary care cooperatives outside a hospital reduce demand for emergency care?. *Emergency Medicine Journal*.

[22] Schuur JD, Venkatesh AK (2012). The growing role of Emergency Departments in hospital admissions. *The New England Journal of Medicine*.

[23] Huibers L, Thijssen W, Koetsenruijter J, Giesen P, Grol R, Wensing M (2013). GP cooperative and emergency department: an exploration of patient flows. *Journal of Evaluation in Clinical Practice*.

[24] Margolius D, Bodenheimer T (2011). Redesigning after-hours primary care. *Annals of Internal Medicine*.

[25] Gaakeer MI, van den Brand CL, Patka P (2012). Emergency medicine in the Netherlands: a short history provides a solid basis for future challenges. *European Journal of Emergency Medicine*.

